# The low indexes of metabolism intervention trial (LIMIT): design and baseline data of a randomized controlled clinical trial to evaluate how alerting primary care teams to low metabolic values, could affect the health of patients aged 75 or older

**DOI:** 10.1186/s12913-017-2812-0

**Published:** 2018-01-05

**Authors:** Nir Tsabar, Yan Press, Johanna Rotman, Bracha Klein, Yonatan Grossman, Maya Vainshtein-Tal, Sophia Eilat-Tsanani

**Affiliations:** 1 Azrieli Faculty of Medicine, Bar-Ilan University, Safed; Unit for Ambulatory Geriatric Services, CHS Northern District, Israel; 2 Unit for Ambulatory Geriatric Services, CHS Southern District and Unit for Community Geriatrics, Division of Health in the Community, Ben-Gurion University of the Negev, Shlomo-Hamelech 17, 8454931 Beer Sheva, Israel; 3 Educational Programs, CHS Northern District, Shadmot Dvora, Israel; 4 Unit for Ambulatory Geriatric Services, CHS Northern District, Migdal Haemek, Israel; 5 Azrieli Faculty of Medicine in Galilee, Bar-Ilan University, Safed; Unit for Home Care, CHS Northern District, Israel; 6 Unit for Clinical Nutrition and Dietetics, CHS Northern District, Afula, Israel; 7 The Department of Family Medicine, Azrieli Faculty of Medicine, Bar-Ilan University, Safed; The Department of Family Medicine, Clalit Health Services, North district, Israel

**Keywords:** Weight loss, Dietetics, Diabetes mellitus, Glycated Hemoglobin A, Cholesterol Deficiency (hypocholesterolemia), Iatrogenic disease, Aged, Aged, 80 and over, Electronic mail, Randomized controlled trial

## Abstract

**Background:**

Too-low body mass index (BMI), HbA1c% or cholesterol levels predicts poor survival. This study investigates whether e-mails about these low values, improve health of people older than 75 years.

**Methods:**

LIMIT - an open label randomized trial - compares usual care to the addition of an e-mail which alerts the family physicians and nurses to low metabolic indexes of a specific patient and advises on nutritional and medical changes. Participants: Clalit Health Services (CHS) patients in the Northern and Southern Districts, aged ≥75 years with any of the following inclusion criteria: a. Significant weight loss: BMI < 23 kg/m^2^ with BMI drop of ≥2 kg/m^2^ during previous two years and without dietitian counseling during previous year. b. Tight diabetic control: HbA1c% ≤ 6.5% and received anti-diabetic medicines during previous 2 months. c. Drug associated hypocholesterolemia: total cholesterol <160 mg/dL and received cholesterol-lowering medicines during previous 2 months. Excluded from criterion c, were patients diagnosed with either ischemic heart disease, transient ischemic attack or stroke. The primary outcome was death from any cause, within one year. In a population of 48,623 people over the age of 75 years, 8584 (17.7%) patients were identified with low metabolic indices and were randomized to intervention or control groups. E-mails were sent on November 2015 to physicians and nurses at 383 clinics.

**Discussion:**

Low metabolic reserve is common in people in Israel’s peripheral districts aged ≥75 years. LIMIT may show whether alerting primary care staff is beneficial.

**Trial registration:**

ClinicalTrials.gov NCT02476578. Registered on June 11, 2015.

**Electronic supplementary material:**

The online version of this article (10.1186/s12913-017-2812-0) contains supplementary material, which is available to authorized users.

## Background

Interactions between diseases, nutritional status and medical treatment become complicated with advancing age. Often, old age is accompanied by multimorbidity as well as increased vulnerability to drug adverse effects [1, 2]. These may result in nutritional imbalance, including malnutrition. Malnutrition is as an important dimension of the elder care quality [3] and is a major cause of vulnerability to stress (frailty) [4].

Malnutrition risk may be monitored by measuring metabolic indexes. Notable examples include Body Mass Index (BMI), and potentially - glycated hemoglobin (HbA1c%) and total serum cholesterol (herein referred to as cholesterol). Many current quality improvement programs monitor metabolic indexes (e.g. [5, 6]). Avoiding high values is the prevalent target while low values are seldom flagged. However, since the correlation of these metabolic indexes with mortality yields a U-shaped curve (see below), low values of these indexes might need some more attention.

Rapid weight loss, especially with low BMI values, usually mandates investigation in order to eliminate the cause or to limit its effect [7, 8]. In a meta-analysis that included 2.88 million people, the optimal BMI for overall survival was found to be 25–30 kg/m^2^ [9] (with a U-shaped curve). However, an optimal individual BMI in the elderly population is a debated issue [10–13]. High-quality nutritional-intervention trials were reported as few and too small in size (26–210 patients each) [14]. These trials used different BMI cut-offs (18.5–24 kg/m^2^) and different weight-loss cut-offs (2.5–10%) as inclusion criteria. Outcomes other than weight change were reported in even fewer trials, which showed trends towards improvements at best. Hence, more research is needed to draw conclusions whether older adults should receive nutritional counseling to avoid weight loss.

The optimal HbA1c% values for survival were 7.5–8% and mortality increased under the 6.5% level among patients who take two antidiabetic medicines [15]. This suggests an increased metabolic deficit risk in tight anti-diabetic phamacological treatment. Since a substantial proportion of older adults with diabetes is potentially overtreated [16], an intervention to reduce overtreatment could be benefitial.

Correlation between death and cholesterol is also U-shaped [17]. Hypocholesterolemia, defined as cholesterol below 160 mg%, predicts increased mortality and morbidity [8, 18]. Clinical questions about statin overtreatment, especially in primary prevention for the elderly, are still unanswered [19]. Prevalence of hyperlipidemia overtreatment, defined as statin treatment of low-risk patients (less than 5% 10-year cardiovascular risk based on the Framingham Heart Study equation) was estimated at 8% [20].

Hence, these low metabolic indexes could potentially be markers for patients that may benefit from appropriate intervention to attenuate the indexes’ decrease. We hypothesized that informational intervention via e-mail to primary physicians and nurses could positively affect the health of these patients. Unfortunately, the scientific evidence regarding e-mail use for clinical communication between healthcare professionals is sparse [21]. While use of computer reminders in family medicine has been used for some decades to effect physician actions (e.g. [22]), we did not find scientific literature on interventions in the community that are similar to our trial (i.e. using e-mail, addressing low metabolic values). However, a hospital based automatic e-mail alert system was found useful in effectively screening patients at risk of malnutrition [23]. Our trial (LIMIT) is thus aimed at investigating the effects of intervening in these identified high risk groups by way of sending one-time e-mail reminders to the primary physician and nurse about their patient’s low metabolic values.

The objectives of LIMIT are shown in Table 1. All outcome measures will be collected from the national CHS computerized database. The primary objective is to determine if the intervention improves patient survival across the groups of high risk patients. The key secondary objectives are to determine if the intervention influences staff response and patient morbidity. Staff response will be assessed by comparing rates of nurse and dietitian evaluations and de-prescribing. Morbidity will be assessed by comparing rates of clinic and emergency-room visits, costs, total drug use, hypoglycemia events etc. The number needed to treat (i.e. number of e-mails sent) to prevent any death in one year will be assessed as a measure of clinical significance [24].

**Table 1:** Outcomes, variables, measures and methods of analysis

Outcome	Hypothesis	Outcome Measure	Analysis Methods
Primary			
Deaths^a^	Intervention decreases overall mortality	All-cause mortality [binary]	Chi-squared test
Secondary			
Nurse evaluation	Intervention enhances rate	Having a nurse evaluation within a year [binary]	Chi-squared test
Dietitian visit in low BMI (<23 kg/m^2^) participants	Intervention enhances rate	Having a dietitian visit within a year [binary]	Chi-squared test
Dispensed anti-diabetic drugs in criterion b patients	Intervention reduces drug-dispensing	WHO DDD of anti-diabetics in first 3 months [continuous]	T-test/ Wilcoxon
Dispensed cholesterol-lowering drugs in criterion c patients	Intervention reduces drug-dispensing	DDD of cholesterol-lowering drugs in first 3 months [continuous]	T-test/ Wilcoxon
Cost	Intervention reduces medical costs	Total CHS expense per patient [continuous]	T-test/ Wilcoxon
Total drug use	Intervention reduces use	Yearly average total DDD [continuous]	T-test/ Wilcoxon
Emergency room visits	Intervention reduces visits	Annual ER visits [continuous]	T-test/ Wilcoxon
BMI - in low BMI (<23 kg/m^2^) participants	Intervention decreases weight-loss	BMI change in a year [continuous]	T-test/ Wilcoxon
HbA1c% - in criterion b patients	Intervention increases HbA1c%	HbA1c in a year [continuous]	T-test/ Wilcoxon
Hypoglycemia - in criterion b patients	Intervention decreases hypoglycemia risk	Number of glucose measures <70 mg/dL [continuous]	T-test/ Wilcoxon
Hypocholesterolemia - in criterion c patients	Intervention decreases hypocholesterolemia	Number of cholesterol measures <160 mg/dL in a year [continuous]	T-test/ Wilcoxon
Subgroup analyses will include			
Tertiles of BMI, HbA1c% and cholesterol	Participants with lower indexes may benefit more		Regression methods with appropriate interaction
Tertiles of Age	Older participants may benefit more		
Gender	A difference may be found		
Prior cardiovascular diagnosis vs. none	A difference may be found		
North vs. South district	Outcomes may be better in the North due to the direct participation of nurses		

*DDD* Defined daily dose

^a^The effect on mortality will also be analyzed for each LIMIT subgroup that has a sufficient number of participants (groups: A, B, C, F; see Table 4)

## Methods/design

LIMIT is a randomized, controlled, open label, superiority trial with 1:1 allocation of two parallel groups. LIMIT is conducted in the CHS community clinics of the Northern and Southern Districts, where CHS service most of the population (71%, 62% respectively), and an even higher percentage within elderly and vulnerable populations. With a 2-year BMI recording rate of 73.2% in our population, a 99.8% rate of recording HbA1c% in patients taking diabetes drugs, and a 99.1% rate of cholesterol level recording, LIMIT is a unique ‘real life’ trial of community medicine. The schedule is presented in Table 2.

**Table 2:** LIMIT schedule of enrolment, intervention and assessment

PERIOD		Enrolment	Allocation	Post-allocation	Close-out
TIME POINT		9–10.2015	19.10.2015	2–9.11.15	1.11.16
ENROLMENT	Eligibility screen	X			
	Informed consent	Not applicable			
	Pre Trial Email	X			
	Randomization		X		
INTERVENTION	Intervention Email			X	
	Control Group				
ASSESSMENT	Primary and Secondary outcomes				X

A pre-trial letter was sent to the primary physicians and nurses in order to introduce the trial protocol and to provide a way to allow them to refrain from participating. Data was extracted from the CHS computerized medical database. Inclusion and exclusion criteria are presented in Table 3.

**Table 3:** LIMIT inclusion and exclusion criteria

To be eligible, a participant must meet inclusion criteria 1,2 AND 3 and not meet the exclusion criterion.
Inclusion Criteria
1. ≥75 years old on 9.2015
2. A member of Clalit Health Services Northern or Southern Districts
3. At risk (one or more of the following):
a. Significant weight loss without dietitian assessment (all the following):
i. A drop in BMI of 2 kg/m2 or more during previous two years
ii. BMI less than 23 kg/m2
iii. No record of dietitian counseling during previous year.
b. Extremely tight medicinal diabetes treatment (both the following):
i. Last HbA1c% ≤ 6.5%
ii. At least one anti-diabetic medicine was dispensed during previous 2 months.
c. Extremely tight lowering of cholesterol (both the following):
i. Last total cholesterol <160 mg/dL (= Hypocholesterolemia)
ii. At least one cholesterol-lowering medicine was dispensed during previous 2 months.
Exclusion criteria
Only for criterion c: Any of the following diagnoses: ischemic heart disease, transient ischemic attack, or stroke.
For all criteria: Patients whose primary clinic staff declined to participate or whose primary clinic staff e-mail address is unobtainable.

Since some participants fit more than one inclusion criterion, 7 subgroups were created, presented in Table 4.

**Table 4:** LIMIT subgroups

Group designation	Criteria	Short description
A	a only	Significant weight loss without dietitian assessment
B	b only	Extremely tight control of diabetes
C	c only	Extremely tight lowering of cholesterol
D	a & b	
E	a & c	
F	b & c	
G	a & b & c	

Randomization was generated by an excel computation (‘RAND’ function) that was repeated automatically until equal numbers of participants (≤ 2% difference^1^) were achieved in all subgroups. The procedure was stopped automatically by using the ‘Goal Seek’ function. The first author activated and recorded the result of the randomization process.

The one-time intervention letter provided relevant patient data and an alert to the primary care providers (physician and nurse) about low values of BMI, HbA1c% or cholesterol with an advice to consider appropriate dietary and medical revision. The intervention e-mails were created by using Microsoft Word ‘Mail Merge’ feature. (Examples are shown in the trial protocol in the Additional file 1). These e-mails were sent automatically using Microsoft Outlook with the ‘Request a Read Receipt’ option checked. Emails were resent, during the first 3 months, only if a “recipient’s mailbox is full” message was received. Open discussions between mail recipients and researchers were encouraged by all means to ensure safety and to add efficacy. All data files and e-mails are stored in secure CHS servers. All trial investigators will have access to the data. The authors adhere to the SPIRIT guidelines for the reporting of trial protocols. The study results will be released to the participating districts’ personnel and to the general medical community. Authorship policy will follow the recommendations of the International Committee of Medical Journal Editors. Regrettably, open data sharing cannot yet be guaranteed.

The intervention arm (patient-specific reminder e-mail letter) will be compared with the control (standard care) for all primary and secondary analyses (Table 1). The proportion of deaths between two groups will be also compared by using a logistic regression model in order to control for confounders: age, gender, BMI, HbA1c%, Cholesterol level, previous MI, IHD, CVA, TIA. Since the primary outcome is death from any cause, we based study power calculation on previously observed mortality differences (1.8% vs. 3%) after e-mailing similar reminders about sulphonyl-urea treatment. Thus, study power of 80% (alpha = 0.01, two-tail Pearson chi-squared test) is achieved by studying 3906 people in each arm. Recruiting 2 CHS districts was needed to reach this number.

Categorical variables will be shown as frequencies and percentages. Continuous variables will be shown using standard distribution indices (e.g. average, standard deviation, median, etc.). Differences between the arms of the study will be examined using Chi-square test (or Fishers’ exact test) for categorical variables, T-test for continuous variables with normal distribution and nonparametric Wilcoxon two sample test for continuous variables without normal distribution. Missing data will be handled using ‘Rubin’s rules’ of multiple imputation and details of the sensitivity analyses will be provided. The mediation of survival differences by the secondary outcomes will be assessed by examining correlation of primary outcome versus secondary outcomes at the subject level, e.g. by performing a logistic regression. The statistical processing will be performed using Excel or SAS 9.2 software and will be statistically significant if *P* < .05 (or *P* < .01 for the primary outcome).

Descriptive statistics are shown in Table 5. Data (up-to-date to 30.9.15) of all 75 years and older members: 26,491 in the Southern district and 22,132 in the Northern district, was collected. For LIMIT’s flow diagram, see Additional file 2.

**Table 5:** Baseline characteristics of the population and of LIMIT participants

Characteristic	All members at age 75+			All LIMIT groups			Group A (BMI criterion a)		
Trial Arm				Total	Email	No Email	Total	Email	No Email
N	48,623			8584	4310	4274	732	370	362
North District	22,132			3490	1774	1716	341	161	180
	(45.5%)			(40.7%)	(41.2%)	(40.1%)	(46.6%)	(43.5%)	(49.7%)
N Gender - Female	28,685			4977	2504	2473	417	210	207
	(59%)			(58%)	(58%)	(58%)	(57%)	(57%)	(57%)
Age [years]	81.6			80.7	80.6	80.8	83.4	82.9	83.8
	(±5.4)			(±4.8)	(±4.7)	(±4.9)	(±5.9)	(±5.7)	(±6.2)
N Age ≥ 90 years	4722			488	232	256	125	54	71
	(9.7%)			(5.7%)	(5.4%)	(6%)	(17.1%)	(14.6%)	(19.6%)
Last BMI [kg/m^2^]	28.5			28.8	28.7	28.8	20.6	20.5	20.6
	(±5.2)			(±5.9)	(±5.9)	(±5.9)	(±2.0)	(±2.0)	(±2.0)
N Last BMI < 20 kg/m^2^	1248			323	149	174	228	122	106
	(2.6%)			(3.8%)	(3.5%)	(4.1%)	(31.1%)	(33%)	(29.3%)
N with Dietitian counseling	1159			240	113	127	0	0	0
	(2.4%)			(2.8%)	(2.6%)	(3%)	(0%)	(0%)	(0%)
HbA1c%	6.3			6.3	6.3	6.3	6.1	6.2	6
	(±1.1)			(±0.9)	(±0.9)	(±0.9)	(±1.1)	(±1.2)	(±0.9)
N HbA1c% ≤ 5.7	12,741			1787	899	888	264	128	136
	(26.2%)			(20.8%)	(20.9%)	(20.8%)	(36.1%)	(34.6%)	(37.6%)
N With DM Medicines	12,131			5566	2771	2795	89	50	39
	(24.9%)			(64.8%)	(64.3%)	(65.4%)	(12.2%)	(13.5%)	(10.8%)
Cholesterol [mg/dL]	177.2			154.3	154.6	154.1	176.2	176.2	176.2
	(±41.9)			(±33.8)	(±33.8)	(±33.8)	(±43.3)	(±43.0)	(±43.6)
N Chol. < 160 mg/dL	17,574			6062	3037	3025	262	125	137
	(36.1%)			(70.6%)	(70.5%)	(70.8%)	(35.8%)	(33.8%)	(37.8%)
N With Cholesterol-lowering Medicines	24,025			6802	3402	3400	228	124	104
	(49.4%)			(79.2%)	(78.9%)	(79.6%)	(31.1%)	(33.5%)	(28.7%)
N s/p MI	8928			1027	489	538	176	99	77
	(18.4%)			(12%)	(11.3%)	(12.6%)	(24%)	(26.8%)	(21.3%)
N s/p CVA/TIA /IHD	22,139			2489	1221	1268	389	210	179
	(45.5%)			(29%)	(28.3%)	(29.7%)	(53.1%)	(56.8%)	(49.4%)
Characteristic	Group B			Group C			Group D		
	(HbA1c criterion b)			(cholesterol criterion c)			(criteria a + b)		
Trial Arm	Total	Email	No Email	Total	Email	No Email	Total	Email	No Email
N	3365	1705	1660	3625	1801	1824	64	33	31
North District	1412	713	699	1417	684	733	32	18	14
	(42%)	(41.8%)	(42.1%)	(39.1%)	(38%)	(40.2%)	(50%)	(54.5%)	(45.2%)
N Gender - Female	1989	999	990	2038	1027	1011	39	19	20
	(59%)	(59%)	(60%)	(56%)	(57%)	(55%)	(61%)	(58%)	(65%)
Age [years]	81	80.9	81.1	80	80	80	80.6	79.9	81.4
	(±4.8)	(±4.6)	(±4.9)	(±4.4)	(±4.4)	(±4.3)	(±5.0)	(±4.3)	(±5.6)
N Age ≥ 90 years	211	103	108	129	66	63	3	1	2
	(6.3%)	(6%)	(6.5%)	(3.6%)	(3.7%)	(3.5%)	(4.7%)	(3%)	(6.5%)
Last BMI [Kg/m^2^]	29.8	29.8	29.7	29.7	29.7	29.7	21	20.9	21.2
	(±5.4)	(±5.4)	(±5.4)	(±5.5)	(±5.4)	(±5.6)	(±1.4)	(±1.5)	(±1.3)
N Last BMI < 20	38	20	18	27	15	12	14	9	5
	(1.1%)	(1.2%)	(1.1%)	(0.7%)	(0.8%)	(0.7%)	(21.9%)	(27.3%)	(16.1%)
N with Dietitian counseling	92	52	40	117	57	60	0	0	0
	(2.7%)	(3%)	(2.4%)	(3.2%)	(3.2%)	(3.3%)	(0%)	(0%)	(0%)
HbA1c%	6.1	6.1	6.1	6.6	6.6	6.6	5.9	6	5.9
	(±0.4)	(±0.4)	(±0.4)	(±1.2)	(±1.2)	(±1.2)	(±0.4)	(±0.4)	(±0.5)
N HbA1c% ≤ 5.7	582	285	297	809	411	398	20	8	12
	(17.3%)	(16.7%)	(17.9%)	(22.3%)	(22.8%)	(21.8%)	(31.3%)	(24.2%)	(38.7%)
N With DM Medicines	3365	1705	1660	1281	621	660	64	33	31
	(100%)	(100%)	(100%)	(35.3%)	(34.5%)	(36.2%)	(100%)	(100%)	(100%)
Chol.[mg/dL]	169.3	169.3	169.3	139.5	139.7	139.3	167.4	168.2	166.5
	(±39.0)	(±39.2)	(±38.8)	(±15.3)	(±14.9)	(±15.7)	(±39.9)	(±41.5)	(±38.1)
N Chol < 160	1365	690	675	3625	1801	1824	24	13	11
	(40.6%)	(40.5%)	(40.7%)	(100%)	(100%)	(100%)	(37.5%)	(39.4%)	(35.5%)
N With Chol.-low Medicines	2120	1057	1063	3625	1801	1824	31	17	14
	(63%)	(62%)	(64%)	(100%)	(100%)	(100%)	(48.4%)	(51.5%)	(45.2%)
N s/p MI	832	430	402	0	0	0	19	9	10
	(24.7%)	(25.2%)	(24.2%)	(0%)	(0%)	(0%)	(29.7%)	(27.3%)	(32.3%)
N s/p CVA/ TIA /IHD	2062	1037	1025	0	0	0	38	21	17
	(61.3%)	(60.8%)	(61.7%)	(0%)	(0%)	(0%)	(59.4%)	(63.6%)	(54.8%)
Characteristic	Group E			Group F			Group G		
	(criteria a + c)			(criteria b + c)			(criteria a + b + c)		
Trial Arm	Total	Email	No Email	Total	Email	No Email	Total	Email	No Email
N	41	21	20	749	376	373	8	4	4
North District	13	9	4	271	129	142	4	2	2
	(31.7%)	(42.9%)	(20%)	(36.2%)	(34.3%)	(38.1%)	(50%)	(50%)	(50%)
N Gender Female	29	13	16	459	233	226	6	3	3
	(71%)	(62%)	(80%)	(61%)	(62%)	(61%)	(75%)	(75%)	(75%)
Age [years]	82.7	83.4	82	79.5	79.2	79.8	79.4	82.8	76
	(±4.5)	(±4.7)	(±4.1)	(±4.1)	(±3.9)	(±4.2)	(±4.5)	(±4.0)	(±1.2)
N Age ≥ 90 years	2	1	1	18	7	11	0	0	0
	(4.9%)	(4.8%)	(5%)	(2.4%)	(1.9%)	(2.9%)	(0%)	(0%)	(0%)
Last BMI [kg/m^2^]	20.8	21	20.6	30.2	30.2	30.2	21.5	21	21.9
	(±1.7)	(±1.7)	(±1.7)	(±5.4)	(±5.7)	(±5.2)	(±1.3)	(±1.7)	(±0.7)
N Last BMI < 20	11	4	7	4	3	1	1	0	1
	(26.8%)	(19%)	(35%)	(0.5%)	(0.8%)	(0.3%)	(12.5%)	(0%)	(25%)
N with Dietitian counseling	0	0	0	31	18	13	0	0	0
	(0%)	(0%)	(0%)	(4.1%)	(4.8%)	(3.5%)	(0%)	(0%)	(0%)
HbA1c%	6.5	6.4	6.7	6.1	6.1	6.1	5.9	6	5.8
	(±1.8)	(±1.4)	(±2.2)	(±0.3)	(±0.3)	(±0.3)	(±0.3)	(±0.2)	(±0.3)
N HbA1c% ≤ 5.7	10	6	4	100	49	51	2	1	1
	(24.4%)	(28.6%)	(20%)	(13.4%)	(13%)	(13.7%)	(25%)	(25%)	(25%)
N With DM Medicines	10	6	4	749	376	373	8	4	4
	(24.4%)	(28.6%)	(20%)	(100%)	(100%)	(100%)	(100%)	(100%)	(100%)
Chol.[mg/dL]	138.5	136.1	141.1	137.4	137.5	137.2	125.1	134.8	115.5
	(±16.2)	(±15.9)	(±16.2)	(±16.1)	(±16.1)	(±16.1)	(±19.6)	(±14.3)	(±19.6)
N Chol < 160	41	21	20	749	376	373	8	4	4
	(100%)	(100%)	(100%)	(100%)	(100%)	(100%)	(100%)	(100%)	(100%)
N With Chol.lowering Medicines	41	21	20	749	376	373	8	4	4
	(100%)	(100%)	(100%)	(100%)	(100%)	(100%)	(100%)	(100%)	(100%)
N s/p MI	0	0	0	0	0	0	0	0	0
	(0%)	(0%)	(0%)	(0%)	(0%)	(0%)	(0%)	(0%)	(0%)
N s/p CVA/ TIA /IHD	0	0	0	0	0	0	0	0	0
	(0%)	(0%)	(0%)	(0%)	(0%)	(0%)	(0%)	(0%)	(0%)

*BMI* Body mass index, *CHS* Clalit health services, *Chol*. Cholesterol, *CVA* Cerebrovascular accident, *DM* Diabetes mellitus, *IHD* Ischemic heart disease, *MI* Myocardial infarct, *N* Number, *s/p* Status post, *TIA* Transient ischemic attack

CHS North and South district 75+ members and LIMIT participants’ data, presented as N (%) or mean (±SD)

BMI < 23 kg/m^2^ was found in 6159 patients (12.7%). BMI drop of at least 2 kg/m^2^ in 2-years was found in 4051 patients (8.3%). Dietitian counseling was reported for 1159 patients (2.4%) during previous year, and only for 38 of the 867 patients who had BMI < 23 kg/m^2^ after losing 2 kg/m^2^. Criterion ‘a’ was met by 845 patients (1.7%).

Diabetes drugs were given to 12,131 patients (25%), of whom (4186, 8.6%) had HbA1c% ≤ 6.5% (criterion b).

Cholesterol lowering drugs were given to 24,013 patients (49.4%). 10,232 of them (21%) had hypocholesterolemia. After exclusion of patients with ischemic cardiac or cerebral diseases, 4423 (9.1%) were included for criterion c.

More than one criterion was met by 862 patients (1.8%). A total of 8584 (17.7%) patients were included and were randomized to intervention or control. Of the included participants, 4977 (58%) were women and 488 (5.7%) were aged 90 years or more.

After randomization, 4310 patient-specific alerts for intervention were prepared by using Mail Merge feature in Microsoft Word. These emails were sent between 2 and 9 November 2015, to 506 physicians and 155 nurses at 383 clinics. Up to 10.12.15 (one month later), 2233 (52%) reading confirmations were received. While sending the intervention e-mails to the physicians and to the Northern district nurses, a concern was raised by some recipients regarding their workload. Hence, the researchers decided to avoid sending the e-mails to the Southern district nurses.

## Discussion

LIMIT was jointly developed by a multi-professional primary-care research team that included family-physicians, geriatricians, nurses and a dietitian. The model for the intervention derived from the currently implemented use of e-mails to alert primary care staff about patient specific ‘quality measures’ focusing on inadequately high indices. A trial addressing malnutrition and drug-overtreatment as new ‘quality measures’ seemed worthwhile.

Regarding the prevalence of LIMIT criteria, a direct comparison with published data is difficult. About 15%–20% elderly patients experience a loss of either 5 kg or more or 5% of usual body weight over 5–10 years and the incidence of unintentional weight loss in studies involving adults seeking health care varies from 1.3% to 8%, depending on the setting and definition of weight loss [25]. Thus, our data may fit within the higher estimations, possibly due to older age. We found no data to compare the rate of dietitian counseling in community weight-losing patients.

We found a similar prevalence of potential overtreatment of diabetes (criterion b) in people aged 65+ in US adults [16]. The baseline prevalence of hypocholesterolemia in our cohort (36% overall and 22% in patients treated with cholesterol-lowering drugs) is higher than estimation based on Lipid Research Clinic Data, 1983 [26] and by Elmehdawi [27] – where hypocholesterolemia prevalence was estimated at 2% to 6% of the elderly. This may reflect differences in cutoff levels and in age of participants (older age comes with wider diversity of measures), a global surge in use of cholesterol-lowering drugs [28] and other reasons.

The high prevalence of elderly people who fit the inclusion criteria, underscores LIMIT’s potential public importance.

## Additional files

Additional file 1: English translation of LIMIT’s protocol. (DOCX 48 kb)

Additional file 2: LIMIT’s - Flow Diagram. (DOCX 30 kb)

## Abbreviations

BMI: Body Mass Index
CHS: Clalit Health Services
HbA1c%: Hemoglobin A1c Percentage
LIMIT: Low Indexes of Metabolism – Intervention Trial

## Footnotes

1 Since one subgroup (E) included 41 participants, a difference of 2.5% (20:21) was specifically allowed.

## References

[1] Routledge PA, O'Mahony MS, Woodhouse KW (2004). Adverse drug reactions in elderly patients. Br J Clin Pharmacol.

[2] Garfinkel D, Mangin D (2010). Feasibility study of a systematic approach for discontinuation of multiple medications in older adults: addressing polypharmacy. Arch Intern Med.

[3] Mamhidir AG, Kihlgren M, Soerlie V. Malnutrition in elder care: qualitative analysis of ethical perceptions of politicians and civil servants. BMC Med Ethics. 2010;11:11–8. doi: 10.1186/1472-6939-11-11. 10.1186/1472-6939-11-11 PMC2927875 20553607

[4] Wells JL, Dumbrell AC (2006). Nnutrition and aging: Assessment and treatment of compromised nutritional status in frail elderly patients. Clin Interv Aging.

[5] U.S. Department of Health and Human Services (2014). 2014 Annual report on the quality of health Care for Adults Enrolled in Medicaid. HHS.

[6] Jaffe DH, Shmueli A, Ben-Yehuda A, Paltiel O, Calderon R, Cohen AD, Matz E, Rosenblum JK, Wilf-Miron R, Manor O (2012). Community healthcare in Israel: quality indicators 2007-2009. Isr J Health Policy Res.

[7] Jack H (2014). Brunner & Suddarth's textbook of medical-surgical nursing.

[8] Wallace JI. Hazzard’s Geriatric Medicine and Gerontology. Halter JB, Ouslander JG, Tinetti ME, Studenski S, High KP, Asthana S, editors 6th ed. New York, NY: McGraw Hill Medical. 2009;469–81.

[9] Flegal KM, Kit BK, Orpana H, Graubard BI. Association of all-cause mortality with overweight and obesity using standard body mass index categories: a systematic review and meta-analysis. JAMA. 2013;309(1):71–82. 10.1001/jama.2012.113905 PMC4855514 23280227

[10] Dixon JB, Egger G, Finkelstein EA, Kral JG, Lambert GW. ‘Obesity Paradox’ misunderstands the biology of optimal weight throughout the life cycle. Int J Obes. 2014;39(1):82–4. 10.1038/ijo.2014.59 24732145

[11] Harrington M, Gibson S, Cottrell RC (2009). A review and meta-analysis of the effect of weight loss on all-cause mortality risk. Nutr Res Rev.

[12] Pan WH, Yeh WT, Chen HJ, Chuang SY, Chang HY, Chen L, Wahlqvist ML (2012). The U-shaped relationship between BMI and all-cause mortality contrasts with a progressive increase in medical expenditure: a prospective cohort study. Asia Pac J Clin Nutr.

[13] Cheng FW, Gao X, Mitchell DC, Wood C, Rolston DD, Still CD, Jensen GL (2016). Metabolic health status and the obesity paradox in older adults. J Nutr Gerontol Geriatr.

[14] MA de van der Schueren, Wijnhoven HA, Kruizenga HM, Visser M. A critical appraisal of nutritional intervention studies in malnourished, community dwelling older persons. Clin Nutr. 2016;35(5):1008–14. 10.1016/j.clnu.2015.12.013 26774525

[15] Currie CJPJ, Tynan A, Evans M, Heine RJ, Bracco OL, Zagar T, Poole CD (2010). Survival as a function of HbA(1c) in people with type 2 diabetes: a retrospective cohort study. Lancet.

[16] Lipska KJ, Ross JS, Miao Y, Shah ND, Lee SJ, Steinman MA (2015). Potential overtreatment of diabetes mellitus in olderd adults with tight glycemic control. JAMA Intern Med.

[17] Iso H, Jacobs DR, Wentworth D, Neaton JD, Cohen JD (1989). Serum cholesterol levels and six-year mortality from stroke in 350,977 men screened for the multiple risk factor intervention trial. N Engl J Med.

[18] Okamura T, Kadowaki T, Hayakawa T, Kita Y, Okayama A, Ueshima H (2003). Nippon Data80 research group: what cause of mortality can we predict by cholesterol screening in the Japanese general population?. J Intern Med.

[19] Gómez-Huelgas R, Giner-Galvañ V, Mostaza J, Cuende J, de Miguel-Yanes J, Rovira E, Sánchez-Fuentes D, Suárez Fernández C, Román Sánchez, P, Group SW. Unanswered clinical questions in the management of cardiometabolic risk in the elderly: a statement of the Spanish society of internal medicine. BMC Cardiovasc Disord. 2014;14:193–9. doi: 10.1186/1471-2261-14-193. 10.1186/1471-2261-14-193 PMC4289584 25519433

[20] Verma A, Visintainer P, Elarabi M, Wartak S, Rothberg M (2012). Overtreatment and undertreatment of hyperlipidemia in the outpatient setting. South Med J.

[21] Goyder C, Atherton H, Car M, Heneghan CJ, Car J. Email for clinical communication between healthcare professionals. Cochrane Database Syst Rev. 2015;2:1–44. 10.1002/14651858.CD007979.pub3 PMC10685995 25698124

[22] Weingarten MA, Bazel D, Shannon HS (1989). Computerized protocol for preventive medicine: a controlled self-audit in family practice. Fam Pract.

[23] Giovannelli J, Coevoet V Vasseur C, Gheysens A, Basse B, Houyengah F. How can screening for malnutrition among hospitalized patients be improved? An automatic e-mail alert system when admitting previously malnourished patients. Clin Nutr. 2014;868–73. 10.1016/j.clnu.2014.09.008 25277380

[24] Cook RJ, Sackett DL (1995). The number needed to treat: a clinically useful measure of treatment effect. BMJ.

[25] Alibhai S, Greenwood C, Payette H. An approach to the management of unintentional weight loss in elderly people. Can Med Assoc J. 2005;172(6):773–80. 10.1503/cmaj.1031527 PMC552892 15767612

[26] Cox RA, García-Palmieri MR (1990). Cholesterol, triglycerides, and associated lipoproteins. Clinical Methods: The History, Physical, and Laboratory Examinations.

[27] Elmehdawi R (2008). Hypolipidemia: a word of caution. Libyan J Med.

[28] Stark Casagrande S, Fradkin JE, Saydah SH, Rust KF, Cowie CC (2013). The prevalence of meeting A1C, blood pressure, and LDL goals among people with diabetes, 1988-2010. Diabetes Care.

[29] U.S. Department of Health and Human Services. Code of Federal Regulations Title 45 Public Welfare Part 46 Protection of Human Subjects. In: HHSgov*.* 2009. http://www.hhs.gov/ohrp/policy/ohrpregulations.pdf. Accessed 12 Apr 2017. 11686173

