# Supplementary material for: The low indexes of metabolism intervention trial (LIMIT): design and baseline data of a randomized controlled clinical trial to evaluate how alerting primary care teams to low metabolic values, could affect the health of patients aged 75 or older
Source: BMC Health Serv Res. 2018 Jan 5;18:4. doi: 10.1186/s12913-017-2812-0 (PMC5755463; doi:10.1186/s12913-017-2812-0)
Supplement: Supplementary file 1 — English translation of LIMIT’s protocol. (DOCX 48 kb) [file 12913_2017_2812_MOESM1_ESM.docx]

Electronic mail to clinic team, advising treatment modifications due to low metabolic indexes of 75+ years old patients: Effect on health measures.

Principal Investigator: Nir Tsabar

Clalit Health Services

POB 131, Bat Hefer 4284200, Israel. Nir.Tsabar@Clalit.Org.IL

*Protocol Version #3 13/10/2015*

*Translated From Hebrew To English*

**Investigators:**

Nir Tsabar, MD, PhD; Yan Press, MD; Bracha Klein, MPA, RN; Yohana Rotman, MPH, RN; Maya Vainshtein Tal, BSc, MHA ,RD; Yonatan Grossman, MD; Sofia Eilat, MD.

**Background:**

Technologic progress promotes large scale medical monitoring and intervention. It enables increased attention to signs of nutritional deficiency. Notable examples include the monitoring of Body Mass Index (BMI), glycated hemoglobin (HbA1c%) and total serum cholesterol (herein referred to as cholesterol), which are integrated in the system of quality measures today (e.g. the Israeli National Program for Quality Indicators in Community, 2012). Monitoring and correcting caloric nutritional deficiencies are critical for optimal medical and nursing care, certainly at ages of 75 years and more (Hazzard's, 2009; Brunner & Suddarth's, 2013).

Interactions between diseases, nutritional status and medical treatment are particularly complicated with advancing age. Old age often comes with multiple morbidity as well as increased vulnerability to medicine adverse effects. These may result in nutritional imbalance, which is detrimental to health and to daily living functions. Malnutrition is a major cause of vulnerability to stress (frailty). Intriguingly, medicines that may harm the digestive system and nutritional status include those that are used frequently to improve 'quality indicators'. Examples include metformin, statins and many more.

Hence, when monitoring health quality indicators, the authors believe it is important to search for optimal values - that include minimum levels (and not only maximum levels) for each physiologic indicator. In this trial, we will focus on BMI, HbA1c% and cholesterol.

BMI is a widely used as an index of nutritional problems. In a meta-analysis which included 2.88 million people, the optimal BMI for overall survival was found to be 25-30 kg/m2 (Flegal, 2013; Dixon, 2014 ; Pan, 2012). Rapid weight loss is a strong indicator for health and functional decline. Such a weight loss, especially with low BMI values, usually mandates investigation in order to eliminate the cause or to limit its effect. A use of an automatic e-mail alert system was found to improve screening rate of malnourished patients in hospital (Giovannelli, 2014).

Correlation between death and HbA1c% is U-shaped, with increased mortality under a 6.5% level in patients taking two antidiabetic medicines (Currie, 2010). Among these patients, the optimal HbA1c% values of for survival were 7.5-8%. A substantial proportion of older adults with diabetes were found by Lipska et al. (2015) to be potentially overtreated.

Correlation between death and cholesterol is also U-shaped (Iso, 1989). Hypocholesterolemia is defined as cholesterol below 160 mg% and predicts increased mortality and morbidity (Hazzard's, p. 1341). We found no interventional study for this situation in the scientific literature.

In our experience, Email alerts to doctors, regarding an over-tight control of specified diabetic patients by sulphonyl-urea medicines was followed by a reduction (from 3% to 1.8%) in mortality comparing to a previous-year matched control group (unpublished observations). While this result was not statistically significant, it encouraged us to try this large scale interventional clinical trial. It also served us in assessing the size of the study population needed.

**Objectives:**

This study aims to determine whether reminding physicians and nurses about too low values of weight (as BMI), glucose (as HbA1c%) or cholesterol (as total serum cholesterol) improves survival and health of people older than 75 years.

**Study Hypotheses:**

A. Main Study Hypotheses:

1. Compliance: Sending the intervention letter will cause a positive change in the relevant index (a rise in BMI, HbA1c%, or cholesterol, respectively).

2. Health effect: Sending the intervention letter will cause a lower mortality rate in each study group compared to control groups, within a year.

B. Other Study Hypotheses: compared to control groups, sending the intervention letter will cause improvement in morbidity and in secondary measures (see below).

And in detail:

Reducing numbers of hospital stays and days of stays, numbers of physician and nurse visits with records of symptoms, number of dispensed medicines, medical expenses; in the HbA1c% intervention - reduced number of dispensed anti-diabetes medicines and reduced rate of measuring glucose levels of 70 mg/dL or less ; in the cholesterol intervention - reduced number of dispensed anti-cholesterol medicines.

Increasing rate of weight measuring, BMI level, HbA1c% measuring, HbA1c% level, cholesterol measuring, cholesterol level; in the BMI intervention - percentage of patients evaluated by a nurse and counseled by a dietician.

**Methodology:**

Briefly: This prospective, randomized controlled trial, will compare BMI, HbA1c% and cholesterol values, rate of death, number of stays, days of stays, numbers of physician and nurse visits with records of symptoms such as "weakness", "dizziness" and total medical expenses of patients 75 years and older that their clinic staff will receive the email concerning them, versus patients that such email concerning them will not be sent. The trial has 3 different alerts: 1. An alert about a significant drop in BMI; 2. An alert about low HbA1c% level in patients treated with diabetes medicines; and 3. An alert about hypocholesterolemia in patients treated with cholesterol-lowering medicines.

Recruiting and inclusion criteria: 75 years and older at the beginning of the trial, clients of Clalit health Services at the North and the South districts, which will be identified thru the computer system (BO) as having one or more of the following:

For alert no. 1: A drop in BMI of 2 Kg/m^2^ or more during the last two years and^[[1]](#footnote-1)^ a BMI lower than 23 Kg/m^2^ and1 no dietician counseling during the last year.

For alert no. 2: HbA1c% of 6.5% or less and1 dispensing diabetes medicines during the last 2 months.

For alert no. 3: Total cholesterol less than 160 mg/dL and1 dispensing cholesterol-lowering medicines during the last 2 months.

Exclusion criteria:

General: Clients that an email address of their physician or nurse is not found.

For alert no. 3: Clients with a diagnosis of myocardial infarction, ischemic heart disease, transient ischemic attack or cortical cerebrovascular accident.

Study schedule:

Sending the alerting mails will be done in one-session during 2015.

Collecting the data will be done after one year.

Publishing the main results will be done within another year at the latest (2017).

Study procedures and methods:

0. Preparations:

A pre-trial email will be sent to the primary physicians on behalf of the district managements in order to inform about the trial. The text is provided in appendix No. 4. Feedback regarding receiving of the letter (full email boxes etc.) will be collected. Primary physicians would be able to choose to refrain from participating.

1. Finding the participants:

A. By using the Clalit health services computer system; an Excel table will be created; which will include these fields: Identity number; Name; Age; Sex; Clinic name; Primary physician name; Primary physician's email address; Nurse in charge at the clinic; email address of that nurse; Last BMI value of the last year; First BMI value of the previous year; Existence of a dietician visit on the last year; Last total cholesterol level; Whether dispensing of a cholesterol lowering medicine was recorded during the last 2 months; Name of (the last) cholesterol lowering medicine dispensed - with dose and quantity; Whether dispensing of a diabetes medicine was recorded during the last 2 months; Name of (the last) diabetes medicine dispensed - with dose and quantity; Whether diagnosed with myocardial infarction; Whether diagnosed with brain infarction; Whether diagnosed with any cardiovascular disease.

B. Participants for each separate inclusion group, according to the criteria above, will be identified. At this stage, a new list will be created, where the people who don't fit any of the criteria will be excluded. Some of the participants may fit more than one criterion. Hence 7 subgroups are created:

A) Patients that fit only alert 1. (Significant weight loss without dietitian assessment)

B) Patients that fit only alert 2. (A potentially too tight medicinal diabetes treatment)

C) Patients that fit only alert 3. (A potentially too tight medicinal cholesterol lowering)

D) Patients that fit both alert 1. and alert 2.

E) Patients that fit both alert 1. and alert 3.

F) Patients that fit both alert 2. and alert 3.

G) Patients that fit all alerts: 1. , 2. and 3.

C. Population size: According to 11.2014 there are about 5000 patients that fit these criteria at North district and a similar number at the South district, totaling ~ 10,000. This provides a sufficient statistical power to examine the effect on the main hypotheses. (We used Pearson **X^2^** test, assuming a 3% to 1.8% decline in death rates, as observed in a similar setting).

2. Randomization:

a. Randomization aims to get an unbiased and equal division into an intervention group and a control group.

b. Randomization will be via computer: by adding a column in the excel table, with the function =ROUND(RAND(),0). This means 1:1 randomization to 0 (control) or 1 (intervention letter).

c. In order to avoid large differences in numbers of participants among the subgroups, the randomization procedure will repeat until the difference between control and intervention group is less than 2%.

3. The alert emails will be prepared and sent via "Mail Merge" (of Microsoft Word) to the primary physicians and to the nurses in charge at the same clinics. The templates to those emails are shown in the appendix. Technical problems (e.g. full mailbox of recipients) will be recorded and managed promptly (- days, hopefully), by the principal investigator.

4. Collecting outcome data: will be via the Clalit health services computer system. Data will include:

a. For all participants (control and trial groups), for the year that follows the intervention email:

1. Mortality (of any cause)

2. Days in stays (of any ward)

3. Number of stays

4. Number of physician visits

5. Number of nurse visits with symptoms

6. Number of all medicines dispensed

7. Medical expenses

8. Last BMI level and date

9. Last cholesterol level and date

10. Last HbA1c% level and date

11. Glucose levels of 70 mg/dL or less, and dates

b. For alert 1., for the year that follows the intervention email:

1. Recording of functional estimation by a nurse

2. Recording nutritional estimation by a nurse

3. Recording dietitian consultation

c. For alert 2. on the 3 months that follows the intervention email:

1. Whether a diabetes medicine was dispensed, and if so:

2. Name of diabetes medicine, dose and quantity

d. For alert 3. on the 3 months that follows the intervention email:

1. Whether a cholesterol lowering medicine was dispensed, and if so:

2. Name of medicine, dose and quantity

5. Data security: will be handled according to Clalit health systems procedures. The information will be saved in allowed secure servers only and will be available to authorized researchers only after their guidance and signing a non-disclosure commitment. When applicable, personal identification details will be omitted from the lists.

6. Outcome analysis:

Categorical variables will be shown as frequencies and percentages. Continuous variables will be shown using standard distribution indices (e.g. average, standard deviation, median, etc.). Differences between the arms of the study will be examined using Chi-square test (or Fishers' exact test) for categorical variables and T-test (or Wilcoxon two sample test) for continuous variables. The statistical processing will be performed using Excel or SAS 9.2 software and will be statistically significant if P <.05 .

7. Ethical issues of the trial:

A waiver of the requirement to obtain a signed informed consent from participants is requested with the following reason: There is no contact with the patients, only unsheathing their existing data.

A waiver of Form 11 is requested with the following reason:

The family physician will be the one who would get the alert related to this trial in the first place.Appendixes:

**Appendix 1. Template for email regarding a patient with BMI <23 Kg/m^2^ that lost ≥2 Kg/m^2^**

Email Subject:

**LIMIT-A trial alert: Signs of metabolic risk for your patient – Mrs./Mr. _________ , I.D. _________**

Email Body:

Dear Dr./Mrs./Mr. **________** ,

This letter is sent to you as a part of an authorized clinical trial. Details about the trial can be found in [this link](https://clinicaltrials.gov/ct2/show/NCT02476578). **Briefly**: Patients aged 75+ with signs of a metabolic risk were found via computer. This detection included **significant weight loss** etc. After randomization to control and intervention groups, relevant alerts are sent for the intervention groups. In this trial we will check the effect of these alerts on treatment, health indexes and survival – a year from now.

Mrs./Mr. **_________** , I.D. **_________**, at **________** clinic of yours, was lately found to have low BMI level ( **____**Kg/m^2^), with a significant drop from previous year level ( **____**Kg/m^2^). We ask you to consider **re-estimation** of his **functional** and **medical** condition and of his treatment, in order to prevent nutritional deterioration, and to consider **referral to dietary counseling**.

We will be grateful for cooperation and will be happy to discuss any questions,

Sincerely,

Dr. Nir Tsabar (Principal investigator)

052-4056670

fax 04-8548890

Nir.Tsabar@Clalit.Org.IL

Further background:

In a meta-analysis which included 2.88 million people, the optimal BMI for overall survival was found to be 25-30 kg/m2 (Flegal, 2013; Dixon, 2014 ; Pan, 2012). Rapid weight loss is a strong indicator for ill health and functional decline. Such a weight loss, especially with low BMI values, usually mandates investigation in order to eliminate the cause or to limit its effect. It might result from a combination of factors, some **preventable**, such as bacterial **infection** (in mouth, stomach etc.), **smoking**, **alcohol** use, **feeding problem**, **constipation**, medicines etc. Commonly used **medicines** that may cause malnutrition include metformin containing tablets, medicines that cause constipation and heartburn (e.g. calcium channel blockers, calcium tablets) and many more.

**Appendix 2. Template for email regarding a patient with low HbA1c% that took an anti-diabetic medicine.**

Email Subject:

**LIMIT-B trial alert: Low HbA1c% level of your patient – Mrs./Mr. _________ , I.D. _________**

Email Body:

Dear Dr./Mrs./Mr. **________** ,

This letter is sent to you as a part of an authorized clinical trial. Details about the trial can be found in [this link](#_top). **Briefly**: Patients aged 75+ with signs of a metabolic risk were found via computer. This detection included **low HbA1c% (<6.5%) with anti-diabetic medicines,** etc. After randomization to control and intervention groups, relevant alerts are sent for the intervention groups. In this trial we will check the effect of these alerts on treatment, health indexes and survival – a year from now.

Mrs./Mr. **_________** , I.D. **_________**, at **________** clinic of yours, was lately found to take anti-diabetic medicine (_______) and the HbA1c% was ____ . This level is low for diabetic medical treatment. According to current guidelines and quality-indicators, a level somewhat below 8% is satisfying and safer.

We ask you to consider **re-estimation** of his functional and medical condition and of his treatment, including **lowering the dose of the anti-diabetic medicine**.

We will be grateful for cooperation and will be happy to discuss any questions,

Sincerely,

Dr. Nir Tsabar (Principal investigator)

052-4056670

fax 04-8548890

Nir.Tsabar@Clalit.Org.IL

Further background:

Correlation between death and HbA1c% is U-shaped, with increased mortality under the level of 6.5% in patients taking two antidiabetic medicines (Currie, 2010). Among these patients, the optimal HbA1c% values of for survival were 7.5-8%. Updated quality-measures consider <8% is satisfying for diabetic patients of age 75+ years.

**Appendix 3. Template for email regarding a patient with low cholesterol that took a cholesterol lowering medicine.**

Email Subject:

**LIMIT-C trial alert: Low cholesterol level of your patient – Mrs./Mr. _________ , I.D. _________**

Email Body:

Dear Dr./Mrs./Mr. **________** ,

This letter is sent to you as a part of an authorized clinical trial. Details about the trial can be found in [this link](#_top). **Briefly**: Patients aged 75+ with signs of a metabolic risk were found via computer. This detection included **low cholesterol (<160mg%) with cholesterol lowering medicines,** etc. After randomization to control and intervention groups, relevant alerts are sent for the intervention groups. In this trial we will check the effect of these alerts on treatment, health indexes and survival – a year from now.

Mrs./Mr. **_________** , I.D. **_________**, at **________** clinic of yours, was lately found to take cholesterol lowering medicine (_______) and the total cholesterol was ____ . This level is low. (Hypocholesterolemia is defined when levels fall below 160mg%.)

We ask you to consider **re-estimation** of his **functional** and **medical** condition and of his treatment, including **lowering the dose of the cholesterol-lowering medicine**.

We will be grateful for cooperation and will be happy to discuss any questions,

Sincerely,

Dr. Nir Tsabar (Principal investigator)

052-4056670

fax 04-8548890

Nir.Tsabar@Clalit.Org.IL

Further background:

In old age, a total cholesterol level of **less** than 160mg% is related to a **greater risk** of functional decline and of death. Cholesterol level may be low due to a combination of factors, including **reversible** ones, such as bacterial **infection**, **smoking**, **malnutrition**, medicines and more. Cholesterol lowering **medicines** may cause nutritional insult and revising their dose and usage is warranted, especially with patients older than 75 years, and particularly if they suffer from possible adverse effects of these medicines. Adverse effects include: musculo-skeletal - pain and gait problem, liver, digestive and respiratory tract symptoms and mental problems such as insomnia, depression etc.

**Appendix 4. A preliminary email from the district medical management to primary physicians**

Subject: Research on "Effect of Electronic mail to clinic team, advising treatment modifications due to low metabolic indexes of 75+ years old patients, on health outcomes."

Dear Doctor,

malnutrition in the elderly is a significant cause of morbidity and mortality.

In the next two months, email letters will be sent to you, as a part of a study that aims to find out whether alerting about low nutritional indexes (weight loss, low HbA1c% or low cholesterol) in elderly patients of yours.

Details of the trial can be found in [this link](#_top).

We ask you to read carefully the emails you'll receive and to relate to the recommendations (of course, according to your professional judgment and to the details of each case).

In case you are not willing to participate in this research - please notify the principal researcher [Dr. Nir Tsabar](mailto:Nir.Tsabar@Clalit.Org.IL), as soon as possible.

Thank you for your cooperation,

Sincerely,

Dr. ________, North/South district medical manager

**Bibliography**

The National Program for Quality Indicators in Community Healthcare in Israel. http://www.health.gov.il/Publicationsfiles/qindicatorsReport2008-2010.pdf Accessed 29 Nov. 2014.

Glucomin patient leaflet. http://www.old.health.gov.il/units/pharmacy/Trufot/ShowAlon.asp?tmpPath=/units/pharmacy/trufot/alonim/Glucomin_pt_minor_changes_1402041326006.pdf Accessed 29 Nov. 2014.

Cresor patient leaflet. http://www.old.health.gov.il/units/pharmacy/Trufot/ShowAlon.asp?tmpPath=/units/pharmacy/trufot/alonim/Crestor_5_10_20_40mg_Tablets_Pt_1405252220492.pdf Accessed 29 Nov. 2014.

Afilalo J, Duque G, Steele R, et al. (2008). Statins for secondary prevention in elderly patients: a hierarchical bayesian meta-analysis. *J Am Coll Cardiol.* 51(1):37-45.

Currie CJ, Peters JR, Tynan A, et al. (2010). Survival as a function of HbA(1c) in people with type 2 diabetes: a retrospective cohort study. *Lancet.* 375(9713):481-9.

Dixon JB, Egger GJ, Finkelstein EA, Kral JG, Lambert GW. (2014). 'Obesity Paradox' misunderstands the biology of optimal weight throughout the life cycle. *Baker International Journal of Obesity.* Apr 15.

Flegal KM, Kit BK, Orpana H, Graubard BI. (2013). Association of all-cause mortality with overweight and obesity using standard body mass index categories: a systematic review and meta-analysis. *JAMA*. 309(1):71-82.

Giovannelli J, Coevoet V, Vasseur C, Gheysens A, Basse B, Houyengah F. (2014). How can screening for malnutrition among hospitalized patients be improved? An automatic e-mail alert system when admitting previously malnourished patients. *Clin Nutr.* Sep 18.

Gutierrez J., Ramirez G., Rundek T., Sacco RL. (2012). Statin Therapy in the Prevention of Recurrent Cardiovascular Events A Sex-Based Meta-analysis. *Arch Intern Med* *, 172* (12), 909-919.

Hinkle J.L., &. C. (2013). *Brunner & Suddarth’s Textbook of Medical-Surgical Nursing* (13 ed.). Philadelphia, Pa: Lippincott, Williams & Wilkins.

Iribarren C., R. D. (1995). Low serum cholesterol and mortality. Which is the cause and which is the effect? *Circulation* *, 92* (9), 2396-403.

Iso H., J. D. (1989). Serum cholesterol levels and six-year mortality from stroke in 350,977 men screened for the multiple risk factor intervention trial. *New England Journal of Medicine* *, 320* (14), 904-10.

Jeffrey Halter, J. O. (2009). *Hazzard's Geriatric Medicine and Gerontology* (6 ed.). New York: McGraw-Hill.

Pan WH, Yeh WT, Chen HJ, Chuang SY, Chang HY, Chen L, Wahlqvist ML. (2012). The U-shaped relationship between BMI and all-cause mortality contrasts with a progressive increase in medical expenditure: a prospective cohort study. *Asia Pac J Clin Nutr.* 21(4):577-87.

Savarese G, Gotto AM Jr, Paolillo S, et al. (2013). Benefits of statins in elderly subjects without established cardiovascular disease: a meta-analysis. *J Am Coll Cardiol*. 62(22):2090-9.

1. AND as intersection [↑](#footnote-ref-1)
