# Supplementary material for: The low indexes of metabolism intervention trial (LIMIT): design and baseline data of a randomized controlled clinical trial to evaluate how alerting primary care teams to low metabolic values, could affect the health of patients aged 75 or older
Source: BMC Health Serv Res. 2018 Jan 5;18:4. doi: 10.1186/s12913-017-2812-0 (PMC5755463; doi:10.1186/s12913-017-2812-0)
Supplement: Supplementary file 2 — LIMIT’s - Flow Diagram. (DOCX 30 kb) [file 12913_2017_2812_MOESM2_ESM.docx]

**LIMIT - LOW INDEXES OF METABOLISM INTERVENTION TRIAL**

**CONSORT 2010 Flow Diagram**

## Planned: Analysis

## Planned: Follow-Up

Allocated to intervention - Email sent (n= **4310**)

♦ Group A (n= 370)

♦ Group B (n= 1705)

♦ Group C (n= 1801)

♦ Group D (n= 33)

♦ Group E (n= 21)

♦ Group F (n= 376)

♦ Group G (n= 4)

## Allocation

Allocated to usual treatment (n= **4274**)

♦ Group A (n= 362)

♦ Group B (n= 1660)

♦ Group C (n= 1824)

♦ Group D (n= 31)

♦ Group E (n= 20)

♦ Group F (n= 373)

♦ Group G (n= 4)

Excluded (n= 40039)

♦ Not meeting inclusion criteria (n= 40039)

## Enrollment

Included (n= **8584**):

♦ Group A (n= 732)

♦ Group B (n= 3365)

♦ Group C (n= 3625)

♦ Group D (n= 64)

♦ Group E (n= 41)

♦ Group F (n= 749)

♦ Group G (n= 8)

Randomized (n= **8584**)

Assessed for eligibility (n=**48623**)
